# Lipidomic Signatures in Pediatric Metabolic Disorders

**DOI:** 10.3390/metabo16010033

**Published:** 2025-12-28

**Authors:** Monica Narvaez-Rivas, Kenneth D. R. Setchell

**Affiliations:** 1 Division of Pathology & Laboratory Medicine, Cincinnati Children’s Hospital Medical Center, Cincinnati, OH 45229, USA; 2 Department of Pediatrics, University of Cincinnati College of Medicine, Cincinnati, OH 45229, USA

**Keywords:** lipidomics, metabolic disorder, children

## Abstract

Lipids are essential biomolecules involved in membrane structure, energy storage, and intracellular signaling. Dysregulation of lipid metabolism (dyslipidemia) plays a central role in a wide spectrum of pediatric metabolic disorders, including both inherited and acquired conditions. Recent and rapid advances in mass spectrometry-based lipidomics have enabled high-resolution profiling of more than one-thousand lipid species, facilitating the discovery of disease-specific lipid signatures that were previously undetectable with conventional biochemical assays. In parallel, the rising prevalence of pediatric obesity, diabetes, asthma, metabolic dysfunction-associated steatotic liver disease (MASLD; formerly referred to as non-alcoholic fatty liver disease or NAFLD) and cancers has accelerated research aimed at uncovering molecular pathways underlying these conditions. Lipidomic approaches have also improved the identification and characterization of rare metabolic disorders. As analytical technologies continue to advance, lipidomics is poised to become a cornerstone of precision medicine in pediatrics, offering new opportunities for early diagnosis, risk stratification, and therapeutic targeting.

## 1. Introduction

Pediatric metabolic disorders (PMDs) are a heterogeneous group of inherited and acquired conditions characterized by disruptions in biochemical pathways essential for energy production, nutrient utilization, and cellular homeostasis [1]. These disorders range from fatty acid oxidation defects and lysosomal storage diseases to obesity, type 1 diabetes (T1D), and MASLD. Because of their early onset and potential progression into adulthood, PMDs require early diagnostic and therapeutic strategies tailored specifically to pediatric physiology [2].

Lipids play indispensable roles in human physiology as structural components of membranes, signaling molecules, and metabolic fuels [3]. In infants and children, lipid metabolism is particularly dynamic due to rapid growth, neurodevelopment, and hormonal changes. Disruptions in lipid homeostasis are increasingly recognized as both markers and mediators of metabolic dysfunction [4]. However, traditional lipid panels have generally focused mainly on cholesterol and triglyceride levels and consequently offer only a limited view of the complex lipid landscape involved in pediatric disease.

Lipidomics—an advanced branch of metabolomics—enables comprehensive profiling of lipid species using liquid chromatography–mass spectrometry (LC–MS), gas chromatography–mass spectrometry (GC–MS), and shotgun lipidomics [5]. These platforms have revolutionized the identification of lipid alterations across a wide array of pediatric disorders [6]. Disease-specific lipidomic signatures have been linked to insulin resistance in obesity [7], altered sphingolipid metabolism in type 1 diabetes [8], and phospholipid abnormalities in rare genetic conditions [9]. Integration with other omics modalities further enriches disease characterization and supports precision medicine [10].

Despite these advances, challenges remain. Pediatric studies often involve small cohorts, ethical constraints, and gaps in age-specific reference ranges [11,12]. Addressing these limitations will be essential for translating lipidomic discoveries into clinical practice.

**Figure 1:**
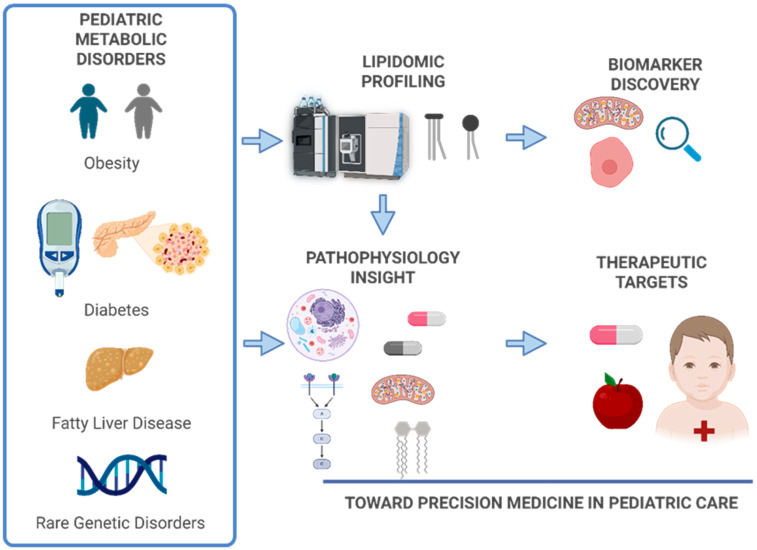
Overview of lipidomic workflow in pediatric populations. The process includes sample collection (e.g., plasma, serum, dried blood spots), lipid extraction using organic solvents, and analysis by mass spectrometry platforms such as liquid chromatography–mass spectrometry (LC–MS) or gas chromatography–mass spectrometry (GC–MS). Data processing involves peak detection, alignment, normalization, and lipid identification using curated databases. Statistical and bioinformatics tools then identify disease-specific lipid signatures, supporting biomarker discovery and integration with other omics for precision medicine. Created in BioRender. Narvaez, M. (Effective Date: 1 October 2025) https://BioRender.com/mrigi2i.

This review synthesizes current knowledge on lipidomic signatures in pediatric metabolic disorders (Figure 1). We discuss lipid physiology in childhood, lipidomic methodologies, disease-specific alterations, and emerging clinical applications, as well as future directions for the field.

## 2. Lipid Metabolism in Pediatric Physiology

### 2.1. Overview of Lipid Roles in Development

During fetal and early postnatal development, lipids supply most of the energy for growth, particularly for the developing brain, which relies heavily on long-chain polyunsaturated fatty acids such as docosahexaenoic acid (DHA) and arachidonic acid (AA) [13]. Breast milk provides a rich source of these essential lipids, and their adequate supply is crucial for neurodevelopment, visual acuity, and immune function [14].

As children grow, the composition and function of lipid classes change [15]. For example, the activity of enzymes involved in lipid synthesis and oxidation, such as lipoprotein lipase, hormone-sensitive lipase, and carnitine palmitoyltransferase, varies with age and nutritional status [16]. These changes influence circulating lipid profiles and tissue lipid composition, which must be considered when evaluating lipidomic signatures in pediatric disorders.

Key lipid classes relevant to pediatric physiology include (summary in Table 1):

- Phospholipids: Major components of cell membranes, essential for maintaining membrane fluidity and facilitating intracellular signaling. These are particularly important during organogenesis and neural development [17].
- Sphingolipids: Involved in cell signaling, membrane stability, and the formation of the myelin sheath. These lipids are critical for proper neurodevelopment and are often implicated in neurological manifestations of metabolic disorders [18].
- Triglycerides: Serve as the primary form of energy storage. In children, triglycerides are mobilized during periods of fasting, illness, and rapid growth to meet increased energy demands [19].
- Cholesterol: Functions as a key structural component of cell membranes and is the precursor for the synthesis of steroid hormones, vitamin D, and bile acids. It plays a vital role in brain development and endocrine function during growth [20,21].
- Fatty Acids: Act as substrates for β-oxidation and are precursors for bioactive lipid mediators such as eicosanoids. Long-chain polyunsaturated fatty acids (LC-PUFAs), including DHA and AA, are essential for brain and retinal development in early life [22].
- Lipoproteins and lipid transport: These are complexes of lipids and proteins that transport hydrophobic lipid molecules through the bloodstream [23]. Their composition and concentration vary with age, sex, and pubertal status [24]. Lipoproteins are crucial for delivering lipids to developing tissues and are increasingly studied as carriers of disease-specific lipidomic signatures in pediatric populations.

All these lipid classes play a role in maintaining cellular and systemic homeostasis, and any dysregulation can contribute to disease pathogenesis.

### 2.2. Age-Related Variability in Lipid Profiles

Pediatric lipid profiles are not static; they change significantly with age, sex, and pubertal status. For instance, total cholesterol and low-density lipoprotein (LDL) levels tend to rise during childhood and peak during adolescence, while high-density lipoprotein (HDL) levels may fluctuate with hormonal changes [25]. These physiological variations complicate the interpretation of lipidomic data and highlight the need for age- and sex-specific reference ranges in pediatric studies [26].

### 2.3. Implications for Lipidomic Research

Given the complexity of lipid metabolism during development, pediatric lipidomic studies must account for physiological variability to avoid misclassification of normal developmental changes as pathological [27]. Longitudinal studies and well-characterized control cohorts are essential for establishing normal ranges and for identifying disease-specific lipidomic alterations.

## 3. Lipidomic Technologies and Methodologies

Lipidomics has rapidly evolved into a powerful analytical discipline, enabling the comprehensive profiling of lipid species in biological systems. The success of lipidomic studies centers on the combination of advanced analytical platforms, meticulous sample preparation, and robust data processing pipelines [28]. This section outlines the main technologies and operational considerations in lipidomic research.

### 3.1. Analytical Platforms

Modern lipidomics is predominantly driven by mass spectrometry (MS)-based technologies, which offer exceptional sensitivity, specificity, and throughput for the analysis of complex lipidomes. Among the most widely utilized platforms is liquid chromatography–mass spectrometry (LC-MS), which couples chromatographic separation with MS detection to enable detailed characterization of diverse lipid species [29]. LC-MS is particularly valued for its versatility and reproducibility, making it suitable for both targeted and untargeted lipidomic analyses. Gas chromatography–mass spectrometry (GC-MS) is another established technique. This is especially effective for the analysis of volatile and derivatized lipids such as fatty acid methyl esters (FAMEs). Its high chromatographic resolution makes it a preferred method for comprehensive fatty acid profiling [30]. A less used method is shotgun lipidomics. This technique consists of a direct infusion MS approach that bypasses chromatographic separation to facilitate rapid, high-throughput lipid profiling. While this method enhances efficiency, it may be limited by ion suppression effects, while the reduced chromatographic resolution makes identification of species more difficult due to the complexity of the data from overlapping closely related lipids in complex biological samples [31]. More recently, emerging techniques such as ion mobility spectrometry (IMS) and four-dimensional lipidomics have been introduced to improve lipid identification. These approaches separate ions based on their shape, size, and charge, thereby increasing confidence in lipid annotation and expanding the analytical depth of lipidomic studies [32].

### 3.2. Sample Preparation

Accurate lipidomic analysis begins with meticulous attention to sample handling and preparation [33], which is particularly critical in pediatric studies where sample volumes are often limited. Common biological matrices include plasma, serum, dried blood spots, and tissue biopsies, each requiring tailored protocols to ensure lipid integrity and reproducibility. Lipid extraction is typically performed using liquid–liquid extraction methods, such as the Bligh and Dyer or Folch protocols, which employ organic solvents—most commonly chloroform, methanol, and water—to efficiently isolate lipids from complex biological matrices. For gas chromatography–mass spectrometry (GC-MS) applications, derivatization steps are often necessary to enhance the volatility and thermal stability of specific lipid classes, such as fatty acids [34]. These preparatory steps are foundational to generating high-quality lipidomic data and ensuring the reliability of downstream analyses.

### 3.3. Data Acquisition and Processing

The transformation of raw MS data into biologically meaningful insights is a multi-step process that requires precision, standardization, and advanced computational tools. This stage is critical for ensuring the accuracy and reproducibility of lipidomic analyses, particularly in pediatric studies where sample volumes are limited and physiological variability may be high.

Following lipid extraction and MS analysis, raw data are generated in the form of spectra that represent the mass-to-charge ratio (m/z) and intensity of detected ions. Key parameters captured during acquisition include retention time (RT), mass to charge ratio (m/z) and ion intensity. High-resolution MS platforms, like Orbitrap and time of flight (TOF) enhance the accuracy of lipid identification by reducing spectral overlap and improving mass accuracy.

Once acquired, raw MS data undergo several computational steps:

1. Peak Detection: Identifies signal peaks corresponding to lipid ions in the spectra.
2. Peak Alignment: Corrects for retention time shifts across samples to ensure consistent comparison.
3. Normalization: Adjusts for technical variability (e.g., batch effects, instrument drift) using internal standards or statistical methods.
4. Lipid Identification: Matches detected features to known lipid species using spectral libraries and databases such as LIPID MAPS (Lipid Metabolites and Pathways Strategy), HMDB (Human Metabolome Database), LipidBlast and MS-DIAL for in silico fragmentation and annotation.
5. Quantification: Can be relative (based on ion intensity) or absolute (using calibration curves and internal standards).

Processed data are subjected to statistical analysis to identify differentially expressed lipids and potential biomarkers. Common approaches include, but are not limited to, univariate analysis (like *t*-tests and ANOVA) for comparing lipid levels between groups, multivariate analysis for pattern recognition (like Principal Component Analysis (PCA), Partial Least Squares Discriminant Analysis (PLS-DA)), and pathway enrichment, which consist of mapping lipid changes to metabolic pathways using tools like MetaboAnalyst or KEGG (Kyoto Encyclopedia of Genes and Genomes) [35]. Meanwhile, MS-DIAL [36] supports deep structural annotation of lipids—including sn-position, double-bond, and hydroxyl-site determination—by integrating electron-activated dissociation data and spatial localization via mass spectrometry imaging with species- and tissue-specific databases. Together with the Integral-Omics workflow, which enables six-layer omics extraction from minute biopsy samples, these tools create a holistic pipeline for comprehensive molecular analysis. This evolving ecosystem allows researchers to streamline sample processing, enhance lipid structural and functional interpretation, and link metabolic alterations to biological pathways—paving the way for early diagnosis, mechanistic insight, and personalized interventions in pediatric metabolic disorders.

These analyses help uncover disease-specific lipid signatures, understand underlying metabolic disruptions, and identify candidate biomarkers for diagnosis or therapeutic targeting.

### 3.4. Strengths and Limitations

These methodologies have different strengths and limitations, which are summarized in Table 2.

Despite these challenges, ongoing advancements in instrumentation, bioinformatics, and standardization are steadily improving the reliability and clinical applicability of lipidomic data.

## 4. Lipidomic Alterations in Common Pediatric Metabolic Disorders

Lipidomics has emerged as a powerful tool for characterizing the molecular foundations of pediatric metabolic disorders. By profiling lipid species across various biological matrices, researchers have identified disease-specific lipid signatures that reflect alterations in lipid metabolism, inflammation, and energy regulation. These insights are particularly valuable in pediatric populations, where early detection and intervention can significantly alter disease trajectories.

### 4.1. Obesity and Metabolic Syndrome

In the last 40 years, obesity in children has increased at least ten-fold worldwide according to the World Health Organization [37]. Childhood obesity is a multifactorial condition characterized by excessive adiposity, often accompanied by insulin resistance, dyslipidemia, and systemic inflammation [38]. The increasing prevalence of pediatric obesity has led to a surge in metabolic syndrome, which is a cluster of conditions including central obesity, hyperglycemia, and dyslipidemia [39]. Lipidomics has provided critical insights into the molecular underpinnings of these conditions, revealing distinct lipid signatures that differentiate metabolically healthy obesity from metabolically unhealthy obesity [7].

Lipidomic studies in obese children have consistently identified elevated saturated and monounsaturated fatty acids, such as palmitic and oleic acids, in serum, which contribute to lipotoxicity and mitochondrial dysfunction, as well as increased ceramides and diacylglycerols, which impair insulin signaling by activating protein kinase C (PKC) pathways, altered phospholipid profiles, including reduced phosphatidylcholine (PC)-to-phosphatidylethanolamine (PE) ratios, indicating compromised membrane integrity and mitochondrial stress, and decreased omega-3 polyunsaturated fatty acids (PUFAs), such as docosahexaenoic acid (DHA), which are anti-inflammatory and neuroprotective. These lipidomic changes are not only biomarkers of metabolic dysfunction but also active contributors to the pathogenesis of insulin resistance and cardiovascular risk.

These findings highlight the complexity of pediatric obesity and emphasize the value of lipidomic profiling in differentiating between low-risk and high-risk metabolic phenotypes. Lipidomic biomarkers can support the early detection of children predisposed to metabolic syndrome, track responses to lifestyle or pharmacological interventions, and enable personalized treatment strategies based on individual metabolic profiles rather than relying solely on clinical measures such as body mass index (BMI).

As lipidomic technologies become more accessible, their integration into pediatric clinical practice could transform the management of obesity-related disorders.

#### 4.1.1. Lipidomic Signatures in Pediatric Obesity

Lipidomic profiling in pediatric obesity has consistently revealed distinct alterations in lipid species that are closely linked to metabolic dysfunction. Among the most frequently observed changes is the elevation of saturated fatty acids (SFAs), particularly palmitic acid [40], which has been implicated in promoting inflammation, endoplasmic reticulum stress, and insulin resistance [41]. In parallel, increased levels of monounsaturated fatty acids (MUFAs), such as oleic acid, have been reported, often reflecting enhanced de novo lipogenesis [42]. Another hallmark finding is the accumulation of ceramides [43], a class of sphingolipids known to disrupt insulin signaling through the activation of protein kinase C (PKC) isoforms and inhibition of Akt phosphorylation [44]. Alterations in phospholipid composition have also been documented [45], including a reduced phosphatidylcholine (PC)-to-phosphatidylethanolamine (PE) ratio, which may indicate compromised membrane integrity and mitochondrial dysfunction [46]. Additionally, a consistent depletion of omega-3 polyunsaturated fatty acids (PUFAs), such as docosahexaenoic acid (DHA), has been observed, potentially diminishing anti-inflammatory and neuroprotective capacities [47].

Recent advances in pediatric lipidomics have highlighted the potential of lipid profiles as early indicators of cardiometabolic risk in obese children. A landmark study [48] examined over 1300 children and adolescents, revealing that obesity is associated with distinct lipidomic alterations—including elevated ceramides, PEs, and triglycerides, alongside reduced omega-3 fatty acids and lysophospholipids. These lipid species correlated strongly with insulin resistance, hypertension, and hepatic steatosis, and a small panel of lipids predicted liver fat as accurately as conventional biomarkers. Importantly, a one-year lifestyle intervention demonstrated that these lipid disturbances are modifiable, underscoring their utility as dynamic biomarkers for risk stratification and therapeutic monitoring in pediatric populations.

These lipidomic signatures not only provide mechanistic insights into the pathophysiology of pediatric obesity but also highlight potential biomarkers for early detection and therapeutic targeting.

#### 4.1.2. Metabolically Healthy vs. Unhealthy Obesity

Several research indicates that obesity does not always lead to metabolic abnormalities or an increased risk of cardiometabolic complications [49,50]. However, due to the absence of universally accepted criteria for defining metabolically healthy obesity (MHO), its reported prevalence varies significantly across studies. Additionally, the long-term health implications of MHO remain controversial, largely because individuals with MHO may gradually transition to metabolically unhealthy obesity (MUO) over time. The concept of MHO versus MUO has gained traction, and lipidomics may offer a resource to distinguish between these phenotypes.

A recent study [49] investigated the plasma metabolome of 311 Asian children and adolescents, comparing those with metabolically healthy obesity (MHO, *n* = 65) and metabolically unhealthy obesity (MUO, *n* = 222) to non-obese controls (*n* = 24). Using untargeted metabolomics, the researchers identified key lipid and metabolite differences between the groups: branched-chain amino acids (BCAAs) and 3-hydroxyisobutyric acid were significantly elevated in MUO compared to MHO, correlating with insulin resistance and impaired glucose tolerance; 1,5-Anhydroglucitol, a marker of short-term glycemic control, was significantly reduced in MUO, indicating poor glucose regulation; plasma fatty acids and lysophospholipids were altered in MUO, suggesting increased lipolysis and inflammation. Multivariate analysis revealed that these metabolomic differences were primarily linked to abnormal glucose homeostasis, highlighting the potential of lipidomics to stratify metabolic risk in obese children.

In another study [50] where metabolic and gut microbial profiles were characterized in Chinese children aged 5–7 years with obesity, LysoPC (O-18:0/0:0), LysoPC (16:1), C16 sphinganine, sphinganine and phytosphingosine were enriched in the MUO group, while PE (16:0/20:4), PI (P-16:0/15:0), and PI (16:0/14:1) were downregulated, suggesting that MUO children may exhibit alterations in ether lipid metabolism and sphingolipid metabolism pathways, showing tentative associations with elevated LysoPCs and reduced PEs/PIs. Therefore, the identification of terpenoid/steroid deficiencies and sphingolipid alterations in MUO children could provide potential biomarkers for distinguishing high-risk subgroups. These findings suggest that lipidomic and metabolomic profiling can stratify metabolic risk in obese children more effectively than anthropometric measures alone.

#### 4.1.3. Clinical Implications and Future Directions

The integration of lipidomics into pediatric obesity research represents a promising advancement in the pursuit of precision medicine. Through the comprehensive analysis of lipid profiles, this approach offers a more detailed characterization of metabolic health than conventional anthropometric measures such as body mass index (BMI). Emerging evidence suggests that lipidomic profiling can facilitate the early identification of children at increased risk for metabolic syndrome, often preceding the onset of overt clinical symptoms. This early detection capability enables timely, targeted interventions that may mitigate the progression to more severe metabolic conditions. Furthermore, lipidomic data provides a dynamic framework for monitoring therapeutic efficacy across a range of interventions, including lifestyle modifications, pharmacologic treatments, and surgical procedures. By capturing biochemical responses in real time, clinicians can refine treatment strategies to enhance responsiveness and adaptability. Importantly, lipidomics supports the development of personalized therapeutic approaches by identifying individual metabolic signatures and lipid pathway disruptions, thereby enabling interventions tailored to the unique biochemical context of each patient. Collectively, these applications underscore the potential of lipidomics to transform clinical management into pediatric obesity through more precise risk stratification, monitoring, and individualized care.

To fully realize the potential of lipidomics in pediatric obesity, future research should prioritize certain studies: (i) longitudinal studies to track lipidomic changes over time may help to elucidate the natural history of metabolic alterations in obese children and identify critical windows for intervention; (ii) development of reference lipid standards to establish age- and sex-specific lipidomic reference ranges, which is essential for accurate interpretation; (iii) clinical application of lipidomic data in pediatric populations; and (iv) integration of Multi-Omics, where lipidomics is combined with other omics platforms—such as genomics, proteomics, and metabolomics to enable the construction of comprehensive metabolic models. These integrative approaches have the potential to uncover novel biomarkers and therapeutic targets, advancing the field toward holistic and system-level understanding of pediatric obesity.

### 4.2. Type 1 and Type 2 Diabetes Mellitus

Lipidomics has emerged as a powerful tool for characterizing metabolic dysregulation in pediatric diabetes, offering insights beyond traditional glycemic markers. Both Type 1 Diabetes Mellitus (T1D) and Type 2 Diabetes Mellitus (T2D) in children exhibit distinct lipidomic profiles that reflect underlying pathophysiological mechanisms and may serve as biomarkers for diagnosis, prognosis, and therapeutic monitoring (Table 3).

Large-scale pediatric T1D studies have significantly advanced the field. For example, the DiPiS cohort [51] analyzed plasma lipidomics in adolescents with genetic risk for T1D and found significant alterations in glycosylated ceramides and long-chain triacylglycerols associated with autoantibody status, reinforcing the role of sphingolipid metabolism in β-cell autoimmunity. Similarly, Liu et al. [52] synthesized data from 18 pediatric cohorts and identified consistent lipidomic alterations preceding clinical onset, including decreased unsaturated triacylglycerols, PCs, and SMs, alongside increased LPCs and branched-chain amino acids (BCAAs). These changes were observed before autoantibody seroconversion, highlighting their predictive value for T1D progression and glycemic control.

Beyond these findings, lipidomic studies in pediatric T1Dconsistently report perturbations in sphingolipid metabolism, particularly increments in ceramide and sphingomyelin species [51,52], which are implicated in β-cell apoptosis and immune modulation [53,54]. Altered levels of lysophosphatidylcholines (LPCs) and phosphatidylethanolamines (PEs) have also been observed [52,55], suggesting disruptions in membrane remodeling and inflammatory signaling [46,56]. These lipid changes may precede clinical onset of symptoms and could serve as early indicators of autoimmune activity or residual β-cell function.

Children with T2D typically exhibit a lipidomic signature characterized by elevated saturated and monounsaturated fatty acids, increased ceramides, and altered acylcarnitine profiles, reflecting insulin resistance and mitochondrial dysfunction [57]. Notably, specific ceramide species (e.g., C16:0 and C18:0) have been associated with hepatic steatosis and cardiovascular risk [58], while reductions in polyunsaturated phospholipids may indicate impaired lipid desaturation and elongation pathways [59,60,61,62,63]. These signatures are often detectable before overt hyperglycemia, highlighting their potential for early risk stratification [64].

While T1D and T2D share some overlapping features, their lipidomic landscapes diverge in meaningful ways. T1D is more closely associated with immune-mediated lipid alterations [54], whereas T2D reflects metabolic overload and lipid accumulation [65]. These differences underscore the utility of lipidomics in distinguishing diabetes subtypes, especially in cases with ambiguous clinical presentation [66].

Therefore, lipidomic profiling offers valuable clinical insights across several dimensions of pediatric diabetes management. First, it can assist in predicting disease progression, as longitudinal alterations in specific lipid species—such as ceramides and acylcarnitines—may reflect worsening insulin resistance or declining β-cell function [67]. Second, it enables monitoring of therapeutic response, given that shifts in lipid profiles often preceded measurable improvements in glycemic control, providing a sensitive and early indicator of intervention efficacy [68]. Finally, lipidomic data could support personalized treatment strategies by identifying individual metabolic signatures that can guide targeted therapies [69], particularly those aimed at modulating ceramide synthesis, fatty acid oxidation, or inflammatory lipid mediators.

To facilitate the clinical translation of lipidomic findings in pediatric diabetes, future research should prioritize several key areas. These include the development of age- and sex-specific reference ranges to ensure accurate interpretation across diverse pediatric populations, and the validation of lipid biomarkers in cohorts representing various diabetes phenotypes and ethnic backgrounds. Additionally, the integration of lipidomics with complementary omics platforms, such as genomics and proteomics, will be essential for constructing robust predictive models of disease onset, progression, and therapeutic response.

By uncovering the lipidomic architecture underlying pediatric diabetes, researchers and clinicians can advance toward mechanism-based, precision approaches to diagnosis, monitoring, and individualized treatment.

### 4.3. Metabolic Dysfunction-Associated Steatotic Liver Disease (MASLD)

Metabolic dysfunction-associated steatotic liver disease (MASLD) has emerged as the most prevalent chronic liver condition in children, particularly among those with obesity and insulin resistance. It encompasses a spectrum of hepatic abnormalities ranging from simple steatosis (fat accumulation) to non-alcoholic steatohepatitis, an inflammatory condition that if untreated leads to hepatocellular injury with progression to fibrosis or cirrhosis.

Lipidomic profiling has uncovered distinct alterations in both hepatic and circulating lipid species that correlate with the severity and progression of pediatric MASLD [70]. Elevated hepatic triglycerides and diacylglycerols are consistently observed and are closely associated with insulin resistance and mitochondrial dysfunction. The increased synthesis of lipogenic fatty acids—particularly palmitate and oleate—reflects activation of de novo lipogenesis (DNL), often driven by hyperinsulinemia and excessive caloric intake. Additionally, MASLD is marked by dysregulated lipoprotein metabolism, including enhanced secretion of very-low-density lipoprotein (VLDL) and reduced levels of high-density lipoprotein (HDL), contributing to systemic dyslipidemia and elevated cardiovascular risk. Ceramide accumulation has also been implicated in hepatic inflammation and insulin resistance, potentially mediating the progression from simple steatosis to steatohepatitis [43]. Collectively, these lipidomic alterations serve not only as biomarkers of hepatic dysfunction but also offer mechanistic insights into the pathophysiology of pediatric MASLD.

Lipidomics offers significant advantages for the clinical evaluation of pediatric MASLD. By detecting disease-specific lipid signatures in plasma or serum, it enables non-invasive differentiation between simple steatosis and non-alcoholic steatohepatitis, reducing reliance on liver biopsy [71,72]. Quantitative lipid profiling further supports disease stratification by assessing severity, predicting progression, and identifying children at risk for fibrosis or cirrhosis [43,73]. In addition, dynamic shifts in lipid species can serve as sensitive biomarkers for monitoring responses to lifestyle modifications, pharmacologic interventions, and emerging treatments targeting lipid metabolism [74].

Combining lipidomics with other omics platforms (e.g., transcriptomics, proteomics) and imaging modalities (e.g., MRI-PDFF, elastography) enhances diagnostic accuracy and provides a systems-level understanding of MASLD. This integrative approach may uncover novel therapeutic targets and support personalized treatment strategies.

In summary, clinical lipidomic signatures of MASLD include hepatic and circulating triglycerides, diacylglycerols, and ceramides, which correlate with insulin resistance and inflammation, as well as dysregulated lipoprotein profiles (increase in VLDL, decrease in HDL) that signal disease severity and cardiovascular risk.

### 4.4. Inborn Errors of Metabolism (IEMs)

Inborn Errors of Metabolism (IEMs) are a heterogeneous group of genetic disorders characterized by defects in specific enzymatic steps within metabolic pathways, leading to the accumulation of substrates or deficiency of essential products. These conditions often present early in life with multisystem involvement, including neurological, hepatic, and skeletal manifestations, and can result in severe morbidity if not promptly diagnosed and treated.

Lipidomics is a powerful tool for characterizing lipid alterations in IEMs, particularly those involving lipid metabolism such as lysosomal storage disorders. Traditional diagnostic approaches have relied on enzyme assays and genetic testing; however, lipidomics provides complementary insights by detecting disease-specific lipid accumulations and secondary metabolic changes. Many IEMs have been associated with abnormal lipid metabolism such as peroxisomal disorders, fatty acid oxidation defects, and cholesterol biosynthesis disorders [75].

Gaucher disease, a lysosomal storage disorder caused by β-glucocerebrosidase deficiency, is characterized by the pathological accumulation of glucosylceramides in macrophages [76]. Lipidomic profiling has proven highly effective in identifying elevated levels of glucosylceramides and glucosylsphingosine (Lyso-GL1), both of which serve as robust biomarkers for disease severity and therapeutic monitoring. Recent studies have demonstrated that Lyso-GL1 concentrations, measured by a targeted approach, correlate with clinical outcomes and can be used to assess the efficacy of enzyme replacement therapy (ERT) and substrate reduction therapy (SRT) in pediatric patients. For instance, Eliglustat-based SRT has shown sustained reductions in Lyso-GL1 levels alongside improved hematologic and visceral parameters in children with Gaucher disease type 1.

Niemann–Pick Disease (Types A and B) is characterized by the pathological accumulation of sphingomyelins and other sphingolipids within lysosomes, resulting from deficient activity of acid sphingomyelinase [77]. Lipidomic analyses in pediatric patients have confirmed significant alterations in lipid profiles, including elevated sphingomyelin species and secondary dyslipidemia marked by low high-density lipoprotein (HDL), high low-density lipoprotein (LDL), and hypertriglyceridemia. These abnormalities correlate with disease phenotype and severity, underscoring the utility of mass spectrometry-based lipidomics for both diagnostic confirmation and monitoring of therapeutic interventions [77].

Fatty acid oxidation defects (FAODs) are a group of inherited metabolic disorders caused by deficiencies in enzymes responsible for mitochondrial β-oxidation. These conditions impair the body’s ability to utilize fatty acids for energy, particularly during fasting or periods of increased energy demand, leading to hypoglycemia, hepatomegaly, cardiomyopathy, and muscle weakness in pediatric patients [75]. Lipidomics provides a powerful approach to the diagnosis of FAOD-related abnormalities by profiling acylcarnitines, free fatty acids, and complex lipid species in plasma or dried blood spots [6]. Mass spectrometry-based lipidomics can identify characteristic patterns such as elevated long-chain acylcarnitines and altered phospholipid composition, which are indicative of defects in enzymes like carnitine palmitoyltransferase (CPT) or very-long-chain acyl-CoA dehydrogenase (VLCAD). Recent studies have demonstrated that untargeted lipidomics can uncover secondary lipid alterations beyond traditional acylcarnitine panels, offering deeper insights into mitochondrial dysfunction and energy metabolism [78]. These findings support the integration of lipidomics into newborn screening and confirmatory diagnostics for FAODs. Early identification of FAODs is critical for preventing metabolic crises and improving outcomes. Lipidomic biomarkers can complement genetic testing and enzyme assays, enabling rapid diagnosis and personalized dietary interventions (e.g., medium-chain triglyceride supplementation).

Peroxisomal disorders, such as Zellweger spectrum disorders (ZSDs) and neonatal adrenoleukodystrophy (NALD), are rare but severe inborn errors of metabolism that manifest early in life and are characterized by defects in peroxisome biogenesis or single peroxisomal enzyme functions [79]. In pediatric patients, these disorders are often present within the first months of life with a constellation of clinical features, including hypotonia, seizures, developmental delay, craniofacial dysmorphisms, hepatomegaly, and sensorineural hearing loss. Zellweger syndrome, the most severe peroxisomal disorders, typically results in death within the first year of life due to progressive multisystem failure. Peroxisomes play a pivotal role in bile acid biosynthesis, particularly in the side-chain oxidation of C27 cholestanoic acid intermediates that are essential for producing the primary C24 bile acids—cholic acid (CA) and chenodeoxycholic acid (CDCA). Two key intermediates in this pathway are 3α,7α-dihydroxy-5β-cholestanoic acid (DHCA) and 3α,7α,12α-trihydroxy-5β-cholestanoic acid (THCA). These long-chain cholestanoic acids tend to accumulate in many, though not all, individuals with peroxisomal disorders, serving as important biochemical indicators of impaired peroxisomal function [80]. At the molecular level, peroxisomal dysfunction leads to the accumulation of very-long-chain fatty acids (VLCFAs), branched-chain fatty acids (e.g., phytanic acid), and bile acid intermediates, alongside deficiencies in plasmalogens, ether phospholipids critical for neural development and membrane integrity [79]. These lipid abnormalities disrupt neuronal myelination, mitochondrial function, and oxidative stress responses, contributing to the profound neurological deterioration observed in affected infants. Lipidomic profiling has emerged as a valuable tool in the early diagnosis and mechanistic understanding of peroxisomal disorders [81]. By enabling the quantification of VLCFAs, plasmalogens, and other peroxisome-related lipid species in plasma or dried blood spots, lipidomics complements traditional biochemical assays and genetic testing [82]. In neonates and infants, where clinical signs may be subtle or nonspecific, lipidomic signatures can facilitate prompt diagnosis, guide confirmatory testing, and inform early therapeutic interventions—such as dietary management bile acid supplementation [80].

Furthermore, longitudinal lipidomic monitoring may offer insights into disease progression and therapeutic efficacy, particularly in milder phenotypes or in patients undergoing experimental treatments. As analytical platforms become more sensitive and pediatric reference ranges are established, lipidomics holds promise for improving the diagnostic yield and clinical management of peroxisomal disorders in early life.

These lipidomic signatures not only confirm diagnosis but also help differentiate between phenotypically similar disorders, reducing diagnostic delays often associated with rare diseases.

Lipidomics plays a critical role in monitoring response to enzyme replacement therapy (ERT) and substrate reduction therapy (SRT) [76]. Quantitative analysis of lipid biomarkers such as Lyso-GL1 in Gaucher disease or sphingomyelin derivatives in Niemann–Pick disease provide real-time feedback on treatment efficacy. Recent studies have demonstrated that ERT in Acid Sphingomyelinase Deficiency (ASMD), namely Olipudase alfa therapy, significantly reduced spleen and liver volumes and normalized lipid profiles in pediatric patients over two years, confirming the utility of lipidomics in long-term monitoring [83]. In the case of SRT in Gaucher Disease, Eliglustat therapy in children showed sustained reductions in glucosylsphingosine levels and improved clinical outcomes, highlighting lipidomics as a sensitive tool for therapeutic assessment [76].

Beyond established conditions, lipidomics facilitates the discovery of novel lipid biomarkers in children with unexplained metabolic symptoms. Integration of lipidomics with genomics and metabolomics accelerates the identification of previously uncharacterized disorders and variants of uncertain significance, improving diagnostic yield and guiding personalized interventions [6].

### 4.5. Rare and Undiagnosed Disorders

In pediatric patients presenting with unexplained metabolic symptoms, lipidomics has emerged as a transformative tool for uncovering rare or previously uncharacterized conditions [6]. By enabling high-resolution profiling of lipid species in biological samples, lipidomics can detect atypical lipid patterns that may not be captured by conventional clinical biochemical assays.

Recent efforts to improve diagnostic accuracy for rare and undiagnosed conditions have leveraged large-scale metabolomic and lipidomic datasets. For example, Kyle et al. [84] developed a comprehensive resource of lipidomic and metabolomic profiles from individuals enrolled in the NIH Undiagnosed Diseases Network. This dataset, which includes plasma, urine, and cerebrospinal fluid samples from patients and healthy controls, captures subtle biochemical alterations that often characterize rare disorders. By making these data publicly available, the study provides a valuable foundation for biomarker discovery and precision medicine approaches, underscoring the potential of lipidomics to identify complex metabolic signatures in cases where conventional diagnostic testing has failed.

In addition to genomic approaches, lipidomics is increasingly recognized as a critical tool for diagnosing rare metabolic disorders. Zandl-Lang et al. [6] argue that while next-generation sequencing has improved gene mutation detection, many cases remain unresolved due to variants of uncertain significance. Lipidomic profiling provides functional insights to identify disease-specific lipid biomarkers that validate these variants and serve as surrogate markers for disease monitoring. The review underscores the importance of mass spectrometry-based lipidomics and multi-omics integration in accelerating biomarker discovery and enabling precision medicine for rare pediatric conditions.

Beyond metabolic disorders, lipidomics is increasingly applied to neurological conditions, including cerebrovascular diseases. Potenza et al. [85] demonstrate how cutting-edge lipidomic technologies have enabled the discovery of unconventional lipid biomarkers in both common and rare cerebrovascular disorders. These biomarkers not only enhance diagnostic accuracy but also provide mechanistic insights and identify novel therapeutic targets, paving the way for personalized medicine approaches in stroke and related conditions.

Mass spectrometry-based omics technologies have significantly advanced rare disease research and diagnostics. These approaches enable comprehensive profiling of proteins, metabolites, and lipids, providing critical insights for biomarker discovery and functional validation of genetic variants [86]. Importantly, MS-based lipidomics and metabolomics have proven particularly valuable for applications such as newborn screening and monitoring therapeutic responses, serving as a powerful complement to genomic data. Collectively, these innovations position mass spectrometry as a cornerstone for accelerating diagnosis, understanding the pathophysiology and implementing precision medicine strategies in rare pediatric conditions. Therefore, as lipidomic databases and analytical tools continue to evolve, their utility in rare disease diagnostics is expected to expand significantly.

## 5. Lipidomics and the Gut–Liver–Brain Axis

The gut–liver–brain axis represents a multidirectional communication network linking the gastrointestinal tract, liver, and central nervous system through metabolic, immune, and neuroendocrine pathways [87]. Lipidomics provides a unique lens to study this axis by identifying lipid species that mediate signaling and metabolic crosstalk.

Gut microbiota profoundly influences host lipid metabolism by producing short-chain fatty acids (SCFAs), modulating bile acid pools, and regulating lipid absorption [88]. SCFAs such as acetate, propionate, and butyrate act as key mediators of gut–liver–brain communication, impacting hepatic lipid oxidation and neuroinflammation through pathways involving PPAR-γ, AMPK, and mTOR signaling [89]. Dysbiosis during early life alters phospholipid and sphingolipid profiles, which can affect immune maturation, metabolic programming, neurodevelopment and stress responses. In pediatric liver disease, microbial metabolites such as deoxycholic acid (DCA) have been linked to disease severity, highlighting the gut microbiota’s role in hepatic lipid remodeling [90].

Lipids act as bioactive messengers within the gut–liver–brain axis. Long-chain polyunsaturated fatty acids (LC-PUFAs), including docosahexaenoic acid (DHA) and arachidonic acid (AA), are essential for synaptic plasticity and cognitive development. Disruptions in these lipids correlate with neurodevelopmental disorders [91]. Similarly, sphingolipids and ceramides link hepatic lipid overload to systemic inflammation and neuronal stress pathways. Recent reviews emphasize that bile acids, acting through the nuclear receptors FXR and TGR5 regulate lipid and glucose metabolism and influence intestinal barrier integrity, further connecting liver and brain health [92].

Integrating lipidomics with microbiome analysis and neuroimaging offers a systems-level approach to understanding the gut–liver–brain axis. Multi-omics strategies combining lipidomics, metabolomics, and metagenomics can identify novel biomarkers for early diagnosis and therapeutic monitoring in pediatric metabolic and neurodevelopmental disorders [93]. These insights pave the way for personalized interventions targeting diet, microbiota modulation, and lipid metabolism.

## 6. Lipidomics and Mental Health in Children

Mental health disorders in children and adolescents—including autism spectrum disorder (ASD), attention-deficit/hyperactivity disorder (ADHD), anxiety, and depression—are increasingly recognized as conditions with metabolic underpinnings. Lipidomics offers a novel approach to understanding these disorders by profiling lipid species involved in neurodevelopment, neurotransmission, and inflammatory signaling.

Lipids play a critical role in brain development, synaptic plasticity, and myelination. Long-chain polyunsaturated fatty acids (LC-PUFAs), such as docosahexaenoic acid (DHA) and arachidonic acid (AA), are essential for neuronal membrane integrity and neurotransmitter function [94]. Lipidomic studies have reported altered phospholipid and sphingolipid profiles in children with ASD and ADHD, suggesting disruptions in membrane composition and signaling pathways that may contribute to cognitive and behavioral symptoms [95,96].

Emerging evidence links dysregulated lipid metabolism to pediatric anxiety and depression [97]. Changes in lysophosphatidylcholines (LPCs), ceramides, and cholesterol esters have been associated with neuroinflammation and impaired neurotransmitter regulation [98]. These lipidomic signatures may serve as biomarkers for early detection and therapeutic monitoring, complementing traditional psychiatric assessments.

Bipolar disorder (BD), although commonly diagnosed during adolescence or adulthood, can also present in childhood and is characterized by severe mood fluctuations and high morbidity. Recent lipidomic research [99] has revealed distinct lipid signatures associated with BD, including reductions in plasmalogens and acyl-carnitines indicative of mitochondrial dysfunction, as well as alterations in phosphatidylcholine and triglyceride species linked to energy metabolism and inflammatory processes. Additionally, disruptions in polyunsaturated fatty acids (PUFAs) and arachidonic acid pathways may influence both disease risk and treatment response [100]. Genomic studies highlight the involvement of the FADS1/2/3 gene cluster, which regulates PUFA metabolism, suggesting that integrating lipidomic and genomic data could enhance biomarker discovery for BD [101]. Furthermore, emerging evidence from gut–brain axis research indicates that microbiota composition may modulate BD pathophysiology and therapeutic outcomes, opening new avenues for microbiome-targeted interventions [102].

Schizophrenia (SCZ) is a severe neuropsychiatric disorder that typically emerges during adolescence and, in rare cases, in childhood. Lipidomic studies have identified profound alterations in lipid metabolism associated with SCZ, including changes in phosphatidylcholine, phosphatidylethanolamine, and triglyceride species that correlate with treatment response in first-episode patients [103]. Dysregulation of sphingolipid and glycerophospholipid pathways—particularly in serum exosomal lipids—has been reported as a potential diagnostic marker with high predictive accuracy (AUC > 0.9) [104]. Additionally, membrane lipid abnormalities, such as reduced sphingomyelin and altered plasmalogen levels, have been linked to cognitive impairment and disruptions in dopamine signaling [105]. Collectively, these findings highlight the promise of lipidomics for identifying biomarkers that enable early diagnosis, predict antipsychotic treatment response, and monitor disease progression in pediatric SCZ.

Integrating lipidomics with microbiome analysis and neuroimaging offers a system-level approach to pediatric mental health. Multi-omics strategies can identify novel biomarkers for risk stratification, guide personalized interventions, and monitor treatment efficacy. Future research should focus on longitudinal studies to validate lipid biomarkers across diverse populations.

## 7. Lipidomics in Pediatric Oncology

Recent studies highlight the clinical promise of lipidomics across pediatric cancers. In acute lymphoblastic leukemia (ALL) [106], LC–MS serum profiling identified 72 significantly altered lipids among 2298 features, with ceramides (Cer 18:0, Cer 20:0) and sphingomyelins most increased; several dysregulated lipids correlated with hematologic indices (↑ leukocytes; ↓ hemoglobin/platelets), supporting their utility as non-invasive biomarkers of disease burden and therapeutic response.

Untargeted plasma lipidomics distinguished medulloblastoma [107] from other intracranial tumors, yielding 97 differential lipids and a seven-lipid diagnostic panel enriched in glycerophospholipid and sphingolipid pathways—pointing to differential diagnosis and risk stratification applications. Additionally, rapid intraoperative classification of pediatric brain tumors [108] (medulloblastoma, pilocytic astrocytoma, ependymoma subtypes) has been demonstrated using 10 s PIRL-MS lipid signatures and a reduced 18-lipid marker array, suggesting a path toward real-time, tissue-sparing decision support during neurosurgery.

Beyond single entities, a recent oncology review [69] synthesizes metabolomic–lipidomic profiling in gliomas, emphasizing recurrent markers (e.g., phosphatidylcholines, sphingomyelins, ceramides, triglycerides) and the translational trajectory from discovery to personalized therapies—a framework relevant to pediatric neuro-oncology as clinical assays mature.

Together, these advances position lipidomics as a complement to genomics/proteomics for non-invasive diagnosis, risk stratification, intraoperative classification, and treatment monitoring across pediatric oncology.

## 8. Conclusions

Lipidomics has emerged as a transformative tool in pediatric medicine, offering unprecedented insights into the molecular underpinnings of metabolic and neurodevelopmental disorders. Lipidomic biomarkers identified across conditions such as obesity, diabetes, MASLD, inborn errors of metabolism, and mental health disorders—including ASD, ADHD, bipolar disorder, and schizophrenia—demonstrate potential for early diagnosis, disease stratification, and therapeutic monitoring. These biomarkers not only reflect pathological lipid remodeling but also provide mechanistic clues that can guide targeted interventions.

From a therapeutic and personalized medicine perspective, lipidomics enables precision approaches by identifying individual metabolic signatures and disrupted lipid pathways. This knowledge supports tailored interventions, ranging from dietary modifications and pharmacologic therapies to even microbiome-targeted strategies. Furthermore, lipidomic profiling offers dynamic monitoring of treatment efficacy, allowing clinicians to adjust therapies in real time and improve patient outcomes.

Recent advances in multiomics integration underscore the importance of comprehensive molecular profiling for precision medicine. The Integral-Omics workflow [109] exemplifies this progress by enabling sequential extraction of metabolites, lipids, genomic DNA, RNA, proteins, and phosphopeptides from biopsy-level tissue samples without reliance on commercial kits or specialized instruments. Benchmarking demonstrated that this approach preserves data quality across all omics layers and is compatible with clinical specimens, as shown in colorectal cancer biopsies where it revealed suppressed ferroptosis pathways and key molecular alterations. By consolidating six omics dimensions within a single workflow, Integral-Omics offers a powerful platform for maximizing information from limited pediatric or rare disease samples, complementing lipidomics and other targeted approaches. Its adaptability and potential for automation position are a transformative tool for future studies aimed at early diagnosis, mechanistic insights, and personalized therapeutic strategies.

Therefore, future efforts should focus on multi-omics integration, robust bioinformatics pipelines, and validation of lipid biomarkers across diverse populations to fully realize the promise of lipidomics in pediatric precision medicine.

## Figures and Tables

**Table 1:** Summary of major lipid classes that are involved in pediatric physiology, their primary biological functions, and their relevance to growth and development in children.

Lipid Class	Primary Functions	Relevance in Pediatric Physiology
**Phospholipids**	Structural components of cell membranes; involved in membrane fluidity and signaling	Crucial for organ development, especially the brain and lungs
**Sphingolipids**	Cell signaling, membrane stability, and myelin sheath formation	Essential for neurodevelopment and nerve conduction
**Triglycerides**	Major energy storage molecules	Provide energy during fasting, illness, and rapid growth phases
**Cholesterol**	Precursor for steroid hormones, vitamin D, and bile acids; membrane structure	Supports hormonal development and digestion; vital for brain and adrenal function
**Fatty Acids**	Energy substrates; precursors for signaling molecules (e.g., eicosanoids)	Long-chain PUFAs (e.g., DHA, AA) are critical for brain and retinal development
**Lipoproteins**	Transport lipids in the bloodstream	Vary with age and puberty; important for lipid delivery to growing tissues

Polyunsaturated fatty acids: PUFAs; Docosahexaenoic acid: DHA; Arachidonic acid: AA.

**Table 2:** Strengths and limitations of methodologies used in lipidomic analysis.

Strengths	Limitations
High sensitivity and specificity	Ion suppression and matrix effects
Broad coverage of lipid classes	Requires extensive standardization
Suitable for both targeted and untargeted analysis	Complex data interpretation
Potential for high-throughput screening	Limited pediatric reference databases

**Table 3:** Comparative Lipidomic Features in Pediatric Type 1 and Type 2 Diabetes Mellitus.

Feature	Type 1 Diabetes (T1D)	Type 2 Diabetes (T2D)
**Pathogenesis**	Autoimmune destruction of pancreatic β-cells	Insulin resistance and β-cell dysfunction, often linked to poor nutrition and obesity
**Key** **Lipidomic** **Changes**	- ↓ Plasmalogens (oxidative stress) - ↑ Sphingomyelins and Ceramides (β-cell apoptosis, immune activation) - ↑ LPCs (inflammation) - Disrupted ether-linked phospholipids	- ↑ TAGs and FFAs (lipolysis, hepatic output) - ↑ Acylcarnitines (mitochondrial overload) - ↑ LPCs and LPEs (inflammation, insulin resistance) - Altered cholesterol esters and phosphatidylinositols
**Biomarker** **Potential**	Early indicators of autoimmune activity and β-cell stress	Correlation with glycemic control, insulin sensitivity, and disease severity
**Clinical** **Implications**	- Early detection of metabolic dysfunction - Monitoring therapeutic response - Identification of novel therapeutic targets	Same as T1D, with emphasis on distinguishing T2D from obesity and predicting rapid progression
**Future** **Directions**	Longitudinal studies, integration with immunologic/genomic data, predictive modeling	Same as T1D

Lysophosphatidylcholines: LPCs; Triacylglycerols: TAGs; Free Fatty Acids: FFAs; Lysophosphatidylethanolamines: LPEs; ↑: increase in concentration; ↓: decrease in concentration.

## Data Availability

No new data were created or analyzed in this study. Data sharing is not applicable to this article.

## Abbreviations

The following abbreviations are used in this manuscript:

PMDs	Pediatric metabolic disorders
T1D	Type 1 diabetes
MASLD	Metabolic dysfunction-associated steatotic liver disease
LC-MS	Liquid chromatography–mass spectrometry
GC-MS	Gas chromatography–mass spectrometry
DHA	Docosahexaenoic acid
AA	Arachidonic acid
LC-PUFAs	Long-chain polyunsaturated fatty acids
LDL	Low-density lipoprotein
HDL	High-density lipoprotein
FAMEs	Fatty acid methyl esters
IMS	Ion mobility spectrometry
RT	Retention time
TOF	Time of flight
HMDB	Human Metabolome Database
PCA	Principal Component Analysis
PLS-DA	Partial Least Squares Discriminant Analysis
KEGG	Kyoto Encyclopedia of Genes and Genomes
PKC	Protein kinase C
PC	Phosphatidylcholine
PE	Phosphatidylethanolamine
BMI	Body mass index
SFAs	Saturated fatty acids
MHO	Metabolically healthy obesity
MUO	Metabolically unhealthy obesity
BCAAs	Branched-chain amino acids
T2D	Type 2 diabetes
DNL	De novo lipogenesis
VLDL	Very-low-density lipoprotein
IEM	Inborn Errors of Metabolism
ERT	Enzyme replacement therapy
SRT	Substrate reduction therapy
FAODs	Fatty acid oxidation defects
CPT	Carnitine palmitoyltransferase
VLCAD	Very-long-chain acyl-CoA dehydrogenase
ZSDs	Zellweger spectrum disorders
NALD	Neonatal adrenoleukodystrophy
CA	Cholic acid
CDCA	Chenodeoxycholic acid
DHCA	3α,7α-dihydroxy-5β-cholestanoic acid
THCA	3α,7α,12α-trihydroxy-5β-cholestanoic acid
VLCFAs	Very-long-chain fatty acids
SCFAs	Short-chain fatty acids
ASD	Autism spectrum disorder
ADHD	Attention-deficit/hyperactivity disorder
BD	Bipolar disorder
SCZ	Schizophrenia
TAGs	Triacylglycerols
